# Swin-GA-RF: genetic algorithm-based Swin Transformer and random forest for enhancing cervical cancer classification

**DOI:** 10.3389/fonc.2024.1392301

**Published:** 2024-07-19

**Authors:** Manal Abdullah Alohali, Nora El-Rashidy, Saad Alaklabi, Hela Elmannai, Saleh Alharbi, Hager Saleh

**Affiliations:** ^1^ Department of Information Systems, College of Computer and Information Sciences, Princess Nourah bint Abdulrahman University, Riyadh, Saudi Arabia; ^2^ Machine Learning and Information Retrieval Department, Faculty of Artificial Intelligence, Kafrelsheiksh University, Kafrelsheiksh, Egypt; ^3^ Department of Computer Science, College of Science and Humanities in Dawadmi, Shaqra University, Shaqra, Saudi Arabia; ^4^ Department of Information Technology, College of Computer and Information Sciences, Princess Nourah bint Abdulrahman University, Riyadh, Saudi Arabia; ^5^ Faculty of Computers and Artificial Intelligence, South Valley University, Hurghada, Egypt; ^6^ Data Science Institute, Galway University, Galway, Ireland; ^7^ Atlantic Technological University, Letterkenny, Ireland

**Keywords:** image processing, image classification, image cancer classification, Swin Transformer, CNN models, genetic algorithm, random forest

## Abstract

Cervical cancer is a prevalent and concerning disease affecting women, with increasing incidence and mortality rates. Early detection plays a crucial role in improving outcomes. Recent advancements in computer vision, particularly the Swin transformer, have shown promising performance in image classification tasks, rivaling or surpassing traditional convolutional neural networks (CNNs). The Swin transformer adopts a hierarchical and efficient approach using shifted windows, enabling the capture of both local and global contextual information in images. In this paper, we propose a novel approach called Swin-GA-RF to enhance the classification performance of cervical cells in Pap smear images. Swin-GA-RF combines the strengths of the Swin transformer, genetic algorithm (GA) feature selection, and the replacement of the softmax layer with a random forest classifier. Our methodology involves extracting feature representations from the Swin transformer, utilizing GA to identify the optimal feature set, and employing random forest as the classification model. Additionally, data augmentation techniques are applied to augment the diversity and quantity of the SIPaKMeD1 cervical cancer image dataset. We compare the performance of the Swin-GA-RF Transformer with pre-trained CNN models using two classes and five classes of cervical cancer classification, employing both Adam and SGD optimizers. The experimental results demonstrate that Swin-GA-RF outperforms other Swin transformers and pre-trained CNN models. When utilizing the Adam optimizer, Swin-GA-RF achieves the highest performance in both binary and five-class classification tasks. Specifically, for binary classification, it achieves an accuracy, precision, recall, and F1-score of 99.012, 99.015, 99.012, and 99.011, respectively. In the five-class classification, it achieves an accuracy, precision, recall, and F1-score of 98.808, 98.812, 98.808, and 98.808, respectively. These results underscore the effectiveness of the Swin-GA-RF approach in cervical cancer classification, demonstrating its potential as a valuable tool for early diagnosis and screening programs.

## 1 Introduction

Cervical cancer poses a significant health burden for women worldwide, with a rising incidence and mortality rate in recent years Beckmann et al. (1). As the fourth most prevalent cancer according to the World Health Organization (WHO), it accounted for 570,000 new cases and over 311,000 deaths in 2018 alone. These alarming statistics can be attributed to various factors, including limited awareness, inadequate early screening options, and a shortage of skilled healthcare professionals Beckmann et al. (1).

Early diagnosis plays a crucial role in improving cervical cancer outcomes and saving lives. Currently, several screening techniques such as visual inspection, Pap tests, histopathology tests, and human papillomavirus (HPV) testing are employed for cervical cancer detection Chitra and Kumar (2). However, manual diagnosis is prone to misdiagnosis due to inter and intra-observer variability. For instance, Pap screening tests require extensive microscopic examinations, which are not only costly but also timeconsuming. Consequently, there is an urgent need to develop an advanced and reliable model capable of providing accurate decision-making in cervical cancer diagnosis Lellée and Küppers (3).

Medical imaging techniques, including magnetic resonance imaging (MRI), computed tomography (CT) scans, and ultrasound, offer detailed insights into infected tissues, and tumor characteristics, and guide treatment decisions such as radiation therapy and chemotherapy Sarhangi et al. (4). The integration of artificial intelligence (AI) with medical imaging has emerged as a promising approach to enhance the accuracy of cervical cancer diagnosis systems Tripathi et al. (5).

Deep learning, a subset of AI, has revolutionized various domains, including computer vision, natural language processing (NLP) Saleh et al. (6), time series analysis Saleh et al. (7), and continuous health monitoring Zhu et al. (8). There is research that applied CNN and pre-trained CNN models to classify cervical cancer. For example, In AlMubarak et al. (9), the authors proposed a hybrid method based on hybrid images and DL models. In Plissiti et al. (10), the authors conducted several experiments using Support Vector Machines, Multi-layer Perceptron (MLP), and CNN. In Alsubai et al. (11), the authors proposed a deep CNN using four convolutional layers. These studies just applied CNN models to classify cervical cancer. These models have limitations that hinder their performance in medical image analysis. These models often focus on local patterns, limiting their ability to grasp the global context of the images Tripathi et al. (5). To overcome these limitations, transfer learning has emerged as a promising approach. Transfer learning leverages pre-trained deep learning models, such as convolutional neural networks (CNNs) Tripathi et al. (5), to transfer learned representations from one task to another, thereby improving classification accuracy. In recent years, researchers have introduced revolutionary approaches like Swin Transformers Cao et al. (12), which utilize shifted windows and self-attention mechanisms to process images hierarchically and efficiently. These transformers excel in capturing both local and global contextual information, exhibiting superior scalability and flexibility compared to traditional CNN models Cao et al. (12). Other research was replace fully connected layers of simple CNN by extreme gradient boosting (XGBoost) to improve classification accuracy of X-ray Images Zivkovic et al. (13).

The main questions of our work are as follows:

- How can we develop an advanced and reliable model that improves the accuracy of cervical cancer diagnosis and overcomes the limitations of existing screening techniques?
- How can we extract local and global features?
- How can we reduce the complicity of feature representation?

To answer these questions, we propose a novel approach, Swin-GA-RF, to enhance the classification performance of cervical cells in Pap smear images. The Swin-GA-RF model combines the strengths of the Swin Transformer, a genetic algorithm (GA) for feature selection, and a random forest classifier. By leveraging the Swin Transformer, we can effectively capture both local and global contextual information from medical images. The GA is then employed to reduce feature complexity and select the most informative features extracted by the Swin Transformer. Finally, the softmax classifier is replaced with a random forest to further enhance the overall classification performance.

The primary contributions of this paper are as follows:

- Introducing the Swin-GA-RF approach, which combines the Swin Transformer, GA-based feature selection, and a random forest classifier for the classification of cervical cells in Pap smear images.
- Leveraging the Swin Transformer to capture both local and global contextual information from medical images, thereby improving the accuracy of classification.
- Employing genetic algorithms to reduce feature complexity and select the most significant features extracted by the Swin Transformer.
- Enhancing the classification performance by replacing the softmax classifier with a random forest.
- Conducting comprehensive comparisons between the proposed Swin-GA-RF model and other models, including Swin Transformer and pre-trained CNN models, using two optimizer methods (SDG and Adam) in both two-class and five-class classification scenarios.
- Demonstrating the superior performance of the proposed model compared to state-of-the-art methods using publicly available cervical cancer datasets.

The remainder of this paper is organized as follows: Section 2 provides a comprehensive review of the relevant literature in the field. In Section 3, we present the methodologies employed, including details about the dataset used and a thorough explanation of the proposed Swin-GA-RF model. The experimental results are discussed in Section 4, followed by the conclusions drawn in Section 6.

## 2 Background literature

The purpose of this section is to review relevant studies on classifying cervical cancer and gaps in the literature.

There are studies have been AI models for classifying cervical cancer.

In AlMubarak et al. (9), the authors proposed a hybrid method based on hybrid images and DL models to classify cervical cancer. The hybrid method improves upon the DL and imaging approaches alone. In Plissiti et al. (10), the authors conducted several experiments using Support Vector Machines, Multi-layer Perceptron (MLP), and CNN to classify cervical cancer. CNN performs better than MLP classifiers. In Alsubai et al. (11), the authors proposed a CNN model using the SIPaKMeD dataset to classify five classes of cervical cells. They used segmented Pap smear images to create augmented images of cervical cells, which were then processed by a deep CNN using four convolutional layers. In Li et al. (14), the authors proposed hybrid models based on CNN and Feature Pyramid Networks (FPN). In addition to the Region Proposal Network (RPN), a global contextual aware module is introduced to improve spatial correlation between the background and the foreground. The results showed that the DGCA-RCNN model achieved the highest performance. In Wu et al. (15), the authors applied CNN with 3012 original images and 108432 augmented images. The results indicated that CNN performed better on original images than on augmented ones. In Manna et al. (16), the authors proposed an ensemble-based model based on fuzzy rank-based fusion using InceptionV3, Xception, and DenseNet 169 to classify two and five classes of cervical cancer. Their proposed model recorded the highest performance. In Pramanik et al. (17), the authors proposed ensemble learning, which integrates the outputs of InceptionV3, MobileNet V2, and Inception ResNet V2. Additionally, they employed fuzzy distance-based measures and applied fuzzy distance measures using the product rule to calculate the final predictions. In Ghoneim et al. (18), the authors employed the proposed CNN-ELM, which is based on convolutional neural networks (CNN) and extreme learning machines (ELM). CNN was utilized for feature extraction, while ELM was employed for classifying normal and abnormal cervical cancer cases. CNN-ELM demonstrated superior performance compared to Multi-Layer Perceptron (MLP) and autoencoder (AE)-based classifiers. In Chen et al. (19), the authors proposed a hybrid loss function with label smoothing to improve the distinguishing power of CNN. The results showed that their proposed model achieved satisfactory accuracy. In Tripathi et al. (5), the authors applied different pre-trained models: InceptionResNetV2, VGG19, DenseNet201, and Xception, to classify cervical images using the SIPaKMeD dataset. In Yaman and Tuncer (20), the authors applied the pre-trained CNN models on the SIPAKMED pap-smear image dataset to the assessment of forthcoming classification techniques. The results showed that ResNet-152 recorded the highest accuracy.

The related work primarily applied DL models and pre-trained CNN models for cervical cancer classification, while others utilized vision transformer models for the same purpose that recorded the lowest performance. In this paper, we propose a novel approach based on Swin Transformer and GA to improve the performance of cervical cell classification. ViT Transformer is employed to capture both local and global contextual information from images, while GA is used to select optimal features from the representations extracted by Swin. Furthermore, the softmax classifier is replaced with a SVM classifier to further enhance performance.

## 3 Materials and method

**Figure 1:**
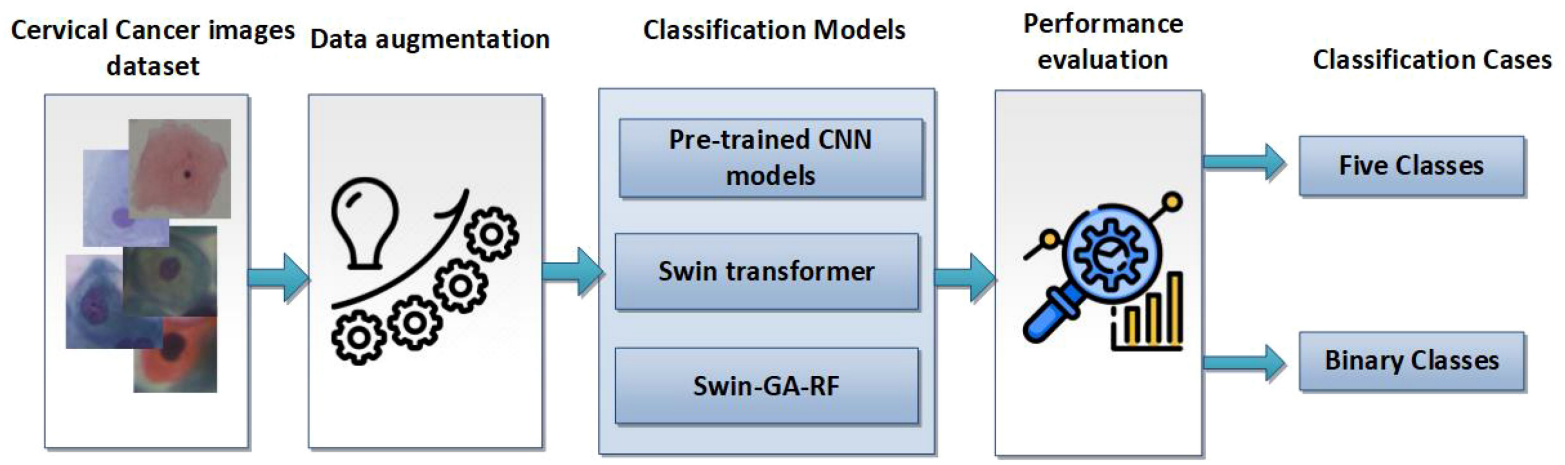
The main steps of classifying cervical cancer detection.

The primary steps involved in classifying cervical cells are depicted in **Figure 1**. The main objective of this study is to propose a novel approach (Swin-GA-RF) aimed at enhancing classification performance by leveraging a combination of techniques, including the Swin Transformer, feature selection, and Random Forest, to classify cervical cells in Pap smear images.

### 3.1 Dataset description

A dataset named SIPaKMeD1, provided by Plissiti et al. dat (2018), is used to evaluate the proposed model (21). It is a balanced dataset and it includes five classes of Pap smear images which are superficial-intermediate (Superf), parabasal (Parab), koilocytes (Koilo), dyskeratotic (Dysker), and metaplastic (Metapl). The dataset includes 3644 images. The number of images in each class is shown in **Table 1**.

**Table 1:** The number of images in each class.

Class	Category	Category	Abbreviation	Totals
1	Abnormal	Dyskeratotic	Dysker	732
2	Abnormal	Koilocytotic	Koilo	742
3	Normal	Metaplastic	Metapl	714
4	Normal	Parabasal	Parab	708
5	Normal	Superficial intermediate	Superf	748

### 3.2 Image augmentation

**Figure 2:**
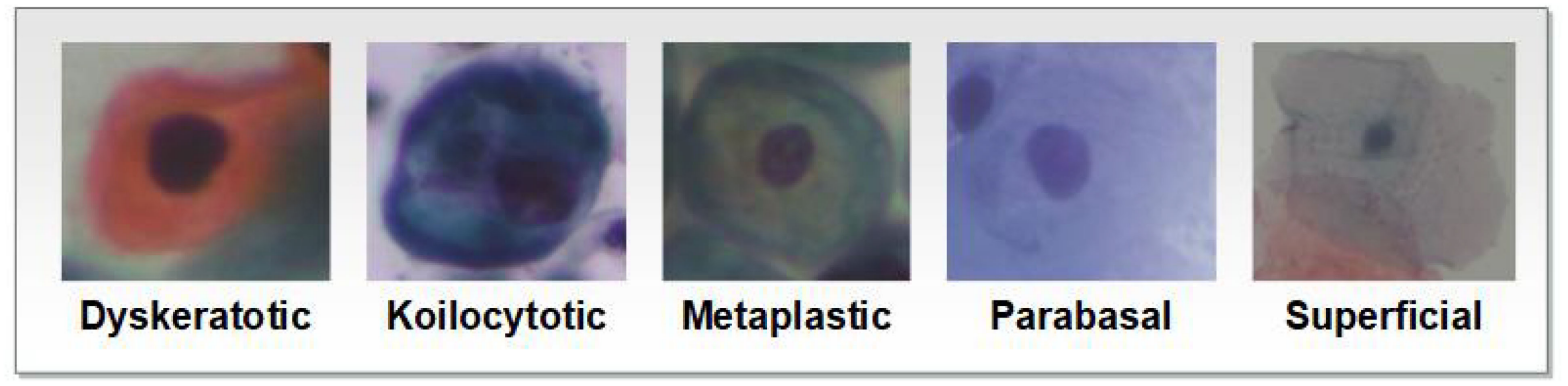
Example of images for SIPaKMeD1.

Image augmentation involves making changes to an image in terms of color and position. Positional manipulation is achieved by altering the position of pixels, while color manipulation involves changing the pixel values. Data augmentation aims to enhance the visual characteristics of an image. It includes techniques such as flipping, cropping, and resizing, Bao et al. (22). These techniques contribute to improving the overall generalization performance of the model by exposing it to a wide variety of images during the training process. Example of images for each class as shown in **Figure 2**.

### 3.3 CNN model

CNN consists of input, an output, and multiple hidden layers.

- The Convolutional layer is the first layer utilized to gather different features, handling the majority of the processing Rai and Rivas (23). Convolution occurs between the input and a window filter by sliding the filter over the input and calculating the dot product over the filter and the input regarding the filter’s size Kuo (24).
- The Pooling layer’s primary goal is to lower the size of the convolved feature map to lower computational expenditures and the number of calculations performed Bailer et al. (25). It summarizes the properties of a convolution layer Nasr-Esfahani et al. (26) The feature map is used to obtain the largest element in max pooling.
- In fully connected layers, each neuron’s input is the weighted sum of all the outputs from the neurons in the layer above Basha et al. (27).

### 3.4 Pre-trained CNN models

DenseNet121, VGG16, ResNet18, and AlexNet are utilized for the classification of cervical cancer.

- DenseNet is a special architecture of the CNN that was developed to address the vanishing gradient problem and improve feature extraction within the network Jaiswal et al. (28).
- ResNet is a residual network that was developed to address the challenges associated with degradation in deep neural networks. ResNet tackles this problem by introducing residual connections, which enable the network to learn residual mappings instead of relying solely on the underlying mappings Deng et al. (29).
- VGG (Visual Geometry Group) is an improved iteration of convolutional neural networks renowned for its effectiveness. The architecture of VGG comprises multiple convolutional layers followed by fully connected layers.
- AlexNet is a deep neural network architecture composed of eight layers, featuring five convolutional layers followed by three fully connected layers. The success of this model can be attributed to several key factors Alzubaidi et al. (30).
- MobileNet is CNN architecture for mobile and embedded devices with minimal computing resources.

These models are tuned for accuracy, minimal computational complexity, and memory footprint Howard et al. (31). Depth-wise separable and point-wise convolutions minimize computational cost in MobileNet models. They are commonly employed in mobile real-time image processing applications Howard et al. (31).

### 3.5 Swin Transformer

Swin Transformer represents a particular type of deep learning model proposed by academics from the Chinese University of Hong Kong and other establishments Liu et al. (32). Swin Transformer’s core principle is to separate the input image into non-overlapping patches and analyze them hierarchically, resulting in a two-stage architecture Liu et al. (33). In the first stage, the model applies self-attention to each local patch to capture local dependencies. In the second stage, the model applies self-attention across patches to capture global dependencies. To handle images with a high resolution efficiently, the Swin Transformer employs a shifted window method Zhang et al. (34). Instead of moving the fixedsized window across the image, the model shifts its position recursively. This enables the model to efficiently capture information at various spatial resolutions Wang et al. (35). The Swin Transformer hierarchical processing mechanism performed well on a variety of computer vision tasks, including picture categorization and object detection, while using fewer computation resources than competing methods Wang et al. (36).

Swin Transformer pairing local self-attention within shifted windows in hierarchical processing to model both local and global relationships in high-resolution images. Input images are first separated into nonoverlapping patch tokens. The patches are then projected linearly into a lower-dimensional embedding space. Swin transforms the patches recursively into shifted windows, allowing the model to gather data at various spatial resolutions Ma et al. (37). The Swin Transformer employs a stack of Transformer encoder blocks. Each encoder block is made up of two major components: a shifting window self-attention module that captures local dependencies between patches inside a window, and a feed-forward neural network that allows the model to record local information within a patch Xiao et al. (38).

The Swin Transformer performs hierarchical processing by stacking several Transformer encoder blocks. The output of one block is used as the input for the next, allowing the model to capture global dependencies across patches Zhou et al. (39). Finally, following hierarchical processing, a classification head is attached to the Swin Transformer to predict class labels for image classification tasks. The classification head is often made up of one or more fully connected layers, which are then activated using softmax Taslimi et al. (40). In general, Swin Transformer has performed well on a variety of image recognition benchmarks, demonstrating its ability to handle enormous amounts of imagery effectively while reaching cutting-edge accuracy and computational efficiency.

**Figure 3:**
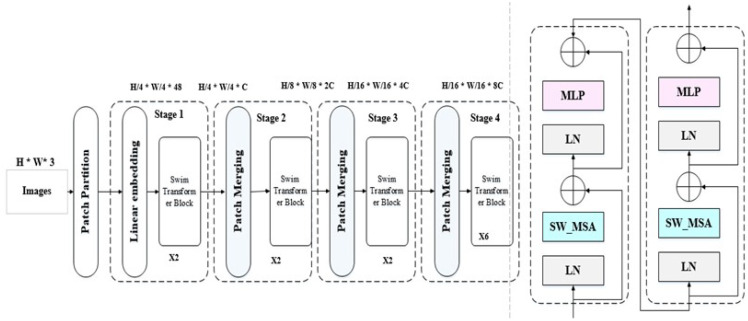
General architecture of swim transformer (32).


**Figure 3** shows the general architecture of the swin transformer. As shown in **Figure 3**, each swim transformer consists of LayerNorm(LN) with multi head attention with 2 MLP layers. Window-based multi-head attention (W MSA) and the shifted Window-based multi-head attention (SW MSA). The swim transformer block could be formulated as follows:

1 $$Z^{l}=W-MSA\;\left(LN\left(Z^{l-1}\right)\right)+Z^{l-1}$$

2 $$Z^{l}=MLP\;(LN\left(Z^{\prime l}\right))+Z^{\prime l}$$

3 $$Z^{\prime +1}=SW-MSA\;\left(LN\left(Z^{l}\right)\right)+Z^{l}$$

4 $$Z^{\prime l+1}=MLP\;\left(LN\left(Z^{\prime l+1}\right)\right)+Z^{\prime l+1}$$

### 3.6 Genetic algorithm

The utilization of Genetic Algorithms (GAs) for feature optimization has been extensively explored across various domains, demonstrating their effectiveness in selecting informative features that yield valuable insights Wang (41). In the context of feature optimization, GAs treat each feature as a gene within the population, forming chromosomes that represent potential solutions comprising specific subsets of features. Evaluating the quality of these solutions is achieved through the fitness function, which assesses their performance based on predefined criteria Reeves (42) El-Rashidy et al. (43).

The GA process initiates by randomly selecting an initial solution from the feature space. Through iterative steps of selection, crossover, and mutation, the process continues, refining the solutions over generations. Solutions with higher fitness values, indicating better performance, have an increased likelihood of being selected for subsequent iterations. GAs have proven to be particularly effective in handling high-dimensional data and supporting multi-objective feature optimization Reeves (42). The strength of GAs lies in their ability to treat each feature as a gene, allowing for comprehensive exploration of the feature space. By iteratively evaluating and evolving solutions through selection, crossover, and mutation, GAs guide the search toward promising subsets of features that improve the classification performance. This capability is especially valuable when dealing with high-dimensional data, enabling the identification of relevant and influential features that contribute to superior classification accuracy Sivanandam et al. (44).

### 3.7 The proposed model

**Figure 4:**
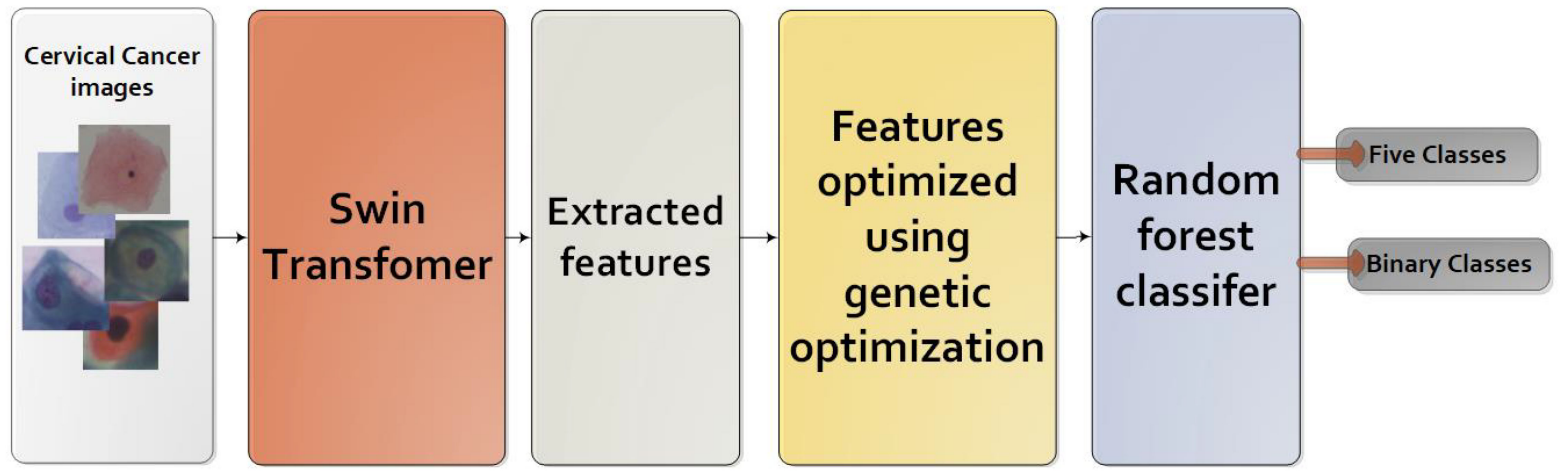
The proposed model.

In our pursuit of building a classifier that achieves exceptional performance, we have devised a groundbreaking model that integrates the Swin transformer, genetic algorithm, and RF. This fusion of techniques allows us to leverage the strengths of each component to create a robust and accurate classifier. The process of constructing this high-performance classifier follows a meticulously designed sequence, encompassing the following steps as shown in **Figure 4**.

- The images are initially divided into small non-overlapping patches, where each patch represents an isolated token.
- All patches are then flattened into 2D tokens, which serve as input for the transformer.
- The Swin transformer incorporates positional information by adding it to the spatial information.
- Window-based positions are utilized to transform the shifted window into the position embedding scheme. This approach enables the Swin transformer to capture both local and global information.
- The Swin transformer processes the aggregated features using stage-wise processing, which includes multiple stages with transformer layers. This strategy enhances processing performance by reducing token resolution in earlier stages and subsequently improving it in subsequent layers.
- Each stage in the transformer consists of multiple layers, with each layer incorporating self-attention and feed-forward layers. The self-attention layers enable the model to capture contextual relationships and relevant patches.
- After processing each window, all tokens are fused to reintegrate the information, facilitating the capture of both local and global information.
- Feature representations are extracted from the layer preceding the softmax layer. Genetic Algorithms (GA) are then applied to reduce feature complexity and select the most significant features. Finally, in the last layer, SoftMax is replaced with a random forest to enhance results.

### 3.8 Evaluation models

The models are evaluated using evaluation metrics, as shown in Equations 1–4. Where True positive (TP)— is the result of the correctly classified positive class. True negative (TN)—the results of the correctly classified negative class. False-positive (FP)—the results of the incorrectly classified positive class. False-negative (FN)—the results of the incorrectly classified negative class.


5 $$Accuracy=\frac{TP+TN}{TP+FP+TN+FN}$$

6 $$Recall=\frac{TP}{TP+FN}$$

7 $$Precision=\frac{TP}{TP+FP}$$

8 $$F1-score=2\cdot \frac{precision\cdot recall}{precision+recall}$$

## 4 Experiments results

This section presents the results of comparing the proposed model (Swin-GA-RF), the Swin Transformer, CNN, and pre-trained CNN models for classifying cervical cells in two classification scenarios: binary and five classes.

### 4.1 Experimental setup

The models were implemented using Python, PyTorch, and the Monai library. The sklearn-genetic library is used to implement genetic feature selection methods Calzolari (45) The image dataset was divided into 70% for training, 10% for validation, and 20% for testing. The number of images in each set is shown in **Table 2**. We conducted experiments using both the Adam and SGD optimizers. The cross-entropy loss function was employed for models. The hyperparameters of Swin, CNN models, and the pre-trained CNN models were CrossEntropy as loss function, epochs=70, and batch size=30. The hyperparameters of RF are Max depth=10, min samples split=2, bootstrap=True, and criterion=gini. A summary of the GA parameters for selecting the best features can be found in **Table 3**.

**Table 2:** The number of images in set.

Class	Category	Category	Abbreviation	Training set (70)	Validation set (10)	Testing set (20)
1	Abnormal	Dyskeratotic	Dysker	569	81	163
2	Abnormal	Koilocytotic	Koilo	577	83	165
3	Normal	Metaplastic	Metapl	555	79	159
4	Normal	Parabasal	Parab	550	79	158
5	Normal	Superficial intermediate	Superf	581	83	167

**Table 3:** Parameters of GA.

Parameters	Values
Crossover rate	0.5
Mutation rate	0.2
Population Size	100
Iteration number	100
population	100

### 4.2 The results of applying models with five classes

#### 4.2.1 The results of Adam optimizer

**Table 4** presents the results of each class obtained from different models, including CNN, DenseNet121, VGG16, ResNet18, AlexNet, Swin Transformer, and Swin-GA-RF using Adam to classify five classes of Cervical Cancer with Adam optimizer. We can see that, CNN records the highest precision, recall, and F1-score at 94.44, 96.84, and 95.62, respectively for the Parab class. CNN records the lowest results for the Koilo and Metapl classes. DenseNet121 consistently demonstrates high precision, recall, and F1-score for Parab, achieving a high recall at 99.37. It particularly excels in the Dysker and Parab classes but struggles with the Koilo class, indicating challenges in accurate classification. VGG16 records the highest precision, recall, and F1-score at 98.70, 96.20, and 97.44, respectively for the Parab class, it faces difficulties in accurately classifying the Koilo class, exhibiting relatively lower precision and recall rates. ResNet18 showcases notable improvements in classification performance, achieving an F1-score of 98.73 for Parab. It demonstrates considerable enhancement in the Dysker, Superf, and Parab classes. However, it encounters challenges in accurately classifying the Koilo class, where its recall rate is comparatively lower. AlexNet records the highest precision, recall, and F1-score at 94.44, 96.84, and 95.62, respectively for the Parab class. The Swin Transformer emerges as a significant improvement over CNN and the pre-trained CNN models, achieving an F1-score of 99.37 for the Parab class. It exhibits significant enhancements in all classes, with notable improvements in the Parab class, where it achieves a precision of 93.90, recall of 93.33, and F1-score of 93.62 with the Koilo class. Finally, the proposed Swin-GA-RF model outperforms all other models, recording the highest overall performance. It records the highest precision of 100, recall of 100, and F1-score of 100 for the Superf class, The Swin-GA-RF model showcases perfect precision, recall, and F1-score, highlighting its ability to accurately classify this type of cervical cancer. These improvements can be attributed to the utilization of the GA for feature selection and the Random Forest classifier, which reduces feature complexity and enhances the overall performance of the model.

**Table 4:** The results of each class for the models to classify five classes of cervical cancer with Adam optimizer.

Models	Classes	Precision	Recall	F1-score
CNN	Dysker	91.72	95.09	93.37
	Koilo	80.95	82.42	81.68
	Metapl	84.39	91.82	87.95
	Parab	94.44	96.84	95.62
	Superf	93.57	78.44	85.34
DenseNet121	Dysker	90.64	95.09	92.81
	Koilo	87.27	87.27	87.27
	Metapl	93.46	89.94	91.67
	Parab	93.45	99.37	96.32
	Superf	96.13	89.22	92.55
VGG16	Dysker	94.44	93.87	94.15
	Koilo	85.37	84.85	85.11
	Metapl	85.55	9308	89.16
	Parab	98.70	96.20	97.44
	Superf	97.48	92.81	95.09
ResNet18	Dysker	96.89	95.71	96.30
	Koilo	93.63	89.09	91.30
	Metapl	91.18	97.48	94.22
	Parab	98.73	98.10	98.41
	Superf	97.01	97.01	97.01
AlexNet	Dysker	95.03	93.87	94.44
	Koilo	88.82	86.67	87.73
	Metapl	89.88	94.97	92.35
	Parab	97.47	97.47	97.47
	Superf	96.34	94.61	95.47
Swin transformer	Dysker	98.11	95.71	96.89
	Koilo	93.90	93.33	93.62
	Metapl	94.55	98.11	96.30
	Parab	99.37	99.37	99.37
	Superf	99.40	98.80	99.10
Swin-GA-RF	Dysker	97.56	98.77	98.16
	Koilo	100	91.57	95.60
	Metapl	95.18	100	97.53
	Parab	97.53	100	98.75
	Superf	100	100	100

**Figure 5:**
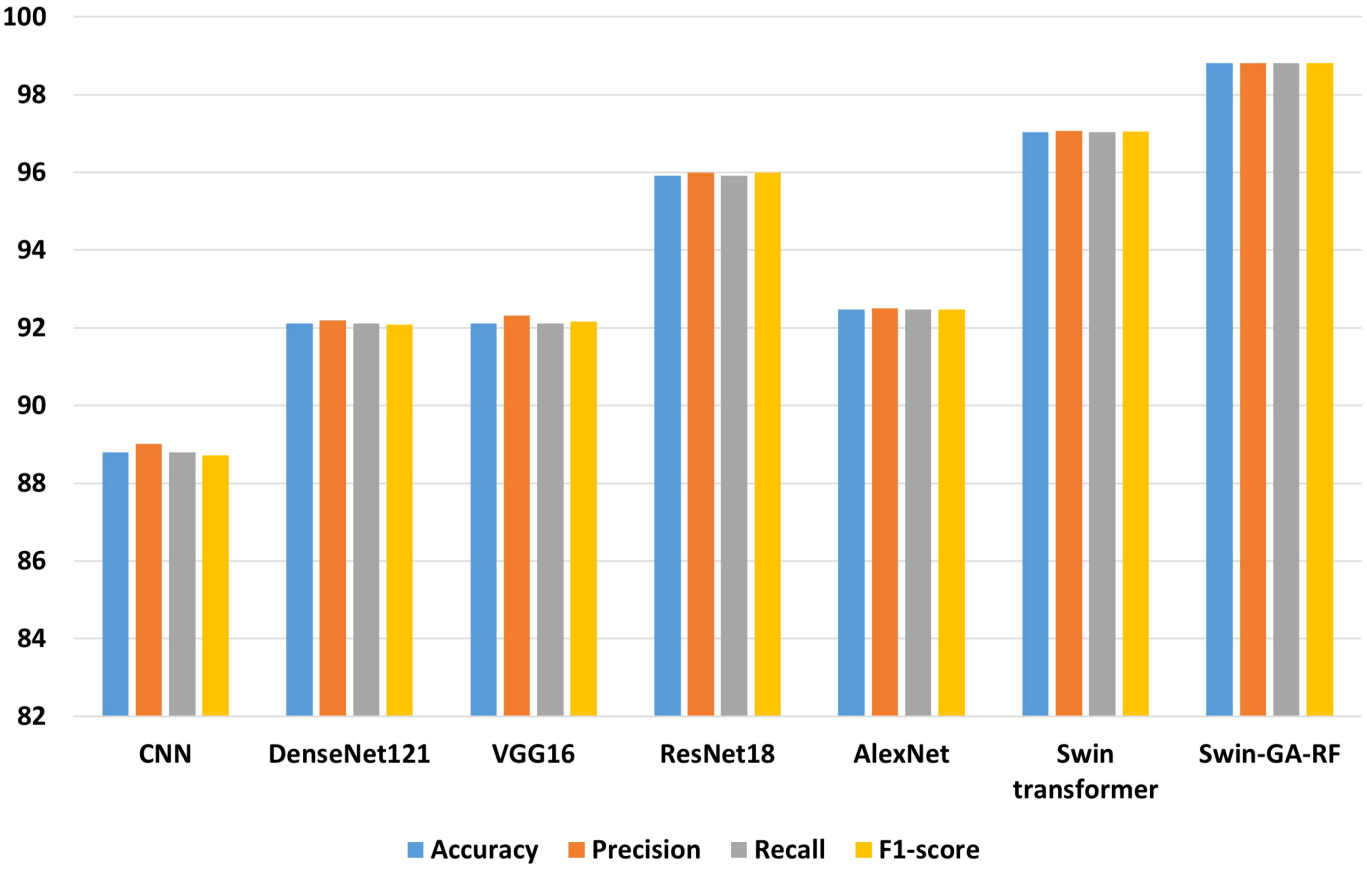
The average of results for models with Adam optimizer for five classes.

**Figure 6:**
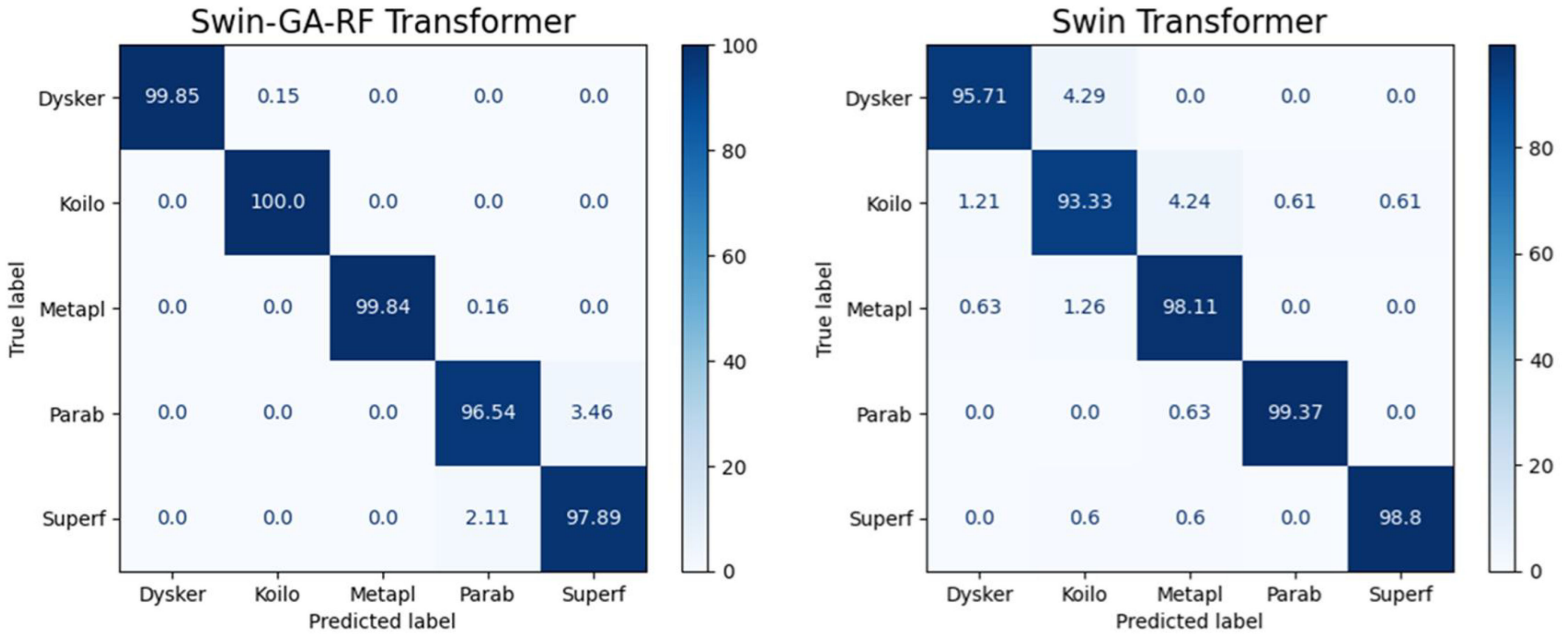
Confusion matrix of Swin and Swin-GA-RF with Adam optimizer for five classes.

**Figure 5** presents the average accuracy, precision, recall, and F1-score of CNN, pre-trained CNN models, Swin Transformer, and Swin-GA-RF models for five classes using Adam optimizer. We can see that the Swin transformer achieves the highest results compared to pre-trained models. Furthermore, the improved version of the Swin transformer, referred to as Swin-GA-RF, records the highest performance with the highest accuracy, F1-score, and improved results at 1.5. This enhancement is attributed to the reduction in feature complexity by the Genetic Algorithm (GA) and Random Forest (RF). Both DenseNet121 and VGG16 exhibit identical results, with accuracy above 92 and recall above 92.308. The third-highest performance is recorded by ResNet18. To compare these results, we conducted a detailed comparison between the Swin transformer and Swin-GA-RF models in each class, as illustrated in **Figure 6**. Swin-GA-RF improved TP by 8% compared to the Swin transformer for the Koilo class.

#### 4.2.2 The results of SGD optimizer

**Table 5** presents the results of each class obtained from different models, including CNN, DenseNet121, VGG16, ResNet18, AlexNet, Swin Transformer, and Swin-GA-RF using SDG to classify five classes of Cervical Cancer with Adam optimizer. CNN records the highest precision, recall, and F1-score at 91.00, 91.09, and 92.60, respectively for class Parab. DenseNet121 consistently demonstrates high precision, recall, and F1-score across various classes, achieving a precision of 97.37 for the Parab class. VGG16 records the highest precision, recall, and F1-score at 91.00, 91.09, and 92.60, respectively for class Parab. ResNet18 showcases notable improvements in classification performance, achieving an average F1-score of 92.57. It demonstrates considerable enhancement in the Dysker and Parab classes, with a precision of 98.70, recall of 96.20, and an F1-score exceeding 97. However, it encounters challenges in accurately classifying the Koilo class, where its recall rate is comparatively lower. VGG16 records the highest precision, recall, and F1-score for the Parab and Superf classes, and the lowest results for Koilo and Metapl classes. ResNet18 showcases notable improvements in classification performance, achieving an F1-score of 98.73 for Parab. AlexNet records the highest precision, recall, and F1-score at 94.44, 96.84, and 95.62, respectively for the Parab class. The Swin Transformer emerges as a significant improvement over CNN and the pre-trained CNN models, achieving an F1-score of 99.37 for the Parab class. It exhibits significant enhancements in all classes, with notable improvements in the Parab class, where it achieves a precision of 93.90, recall of 93.33, and F1-score of 93.62 with the Koilo class. Finally, the proposed Swin-GA-RF model outperforms all other models, recording the highest overall performance. It achieves notable improvements in the Dysker, Koilo, and Metapl classes, demonstrating enhanced precision, recall, and F1-score compared to the Swin Transformer. The Swin-GA-RF model showcases perfect precision, recall, and F1-score in the Parab class, highlighting its ability to accurately classify this type of cervical cancer. These improvements can be attributed to the utilization of the Genetic Algorithm (GA) for feature selection and the Random Forest classifier, which reduces feature complexity and enhances the overall performance of the model.

**Table 5:** The results of each class for the models to classify five classes of cervical cancer with SDG optimizer.

Models	Classes	Precision	Recall	F1-score
CNN	Dysker	89.11	89.57	89.13
	Koilo	85.16	85.88	85.49
	Metapl	83.48	80.38	80.07
	Parab	91.00	91.09	92.6
	Superf	87.19	88.62	88.39
DenseNet121	Dysker	93.90	94.48	94.19
	Koilo	87.01	81.21	84.01
	Metapl	87.04	88.68	87.85
	Parab	96.88	98.10	97.48
	Superf	88.95	91.62	90.27
VGG16	Dysker	94.48	94.48	94.48
	Koilo	89.33	81.21	85.08
	Metapl	83.89	94.97	89.09
	Parab	97.37	93.67	95.48
	Superf	96.41	96.41	96.41
ResNet18	Dysker	95.97	98.16	97.56
	Koilo	95.71	87.09	90.74
	Metapl	91.18	95.48	92.22
	Parab	99.00	98.73	99.36
	Superf	97.22	97.40	97.81
AlexNet	Dysker	93.45	96.32	94.86
	Koilo	88.96	87.88	88.41
	Metapl	88.55	92.45	90.46
	Parab	97.35	93.04	95.15
	Superf	96.95	95.21	96.07
Swin transformer	Dysker	97.53	96.93	97.23
	Koilo	92.77	93.33	93.05
	Metapl	96.20	95.60	95.90
	Parab	98.11	98.11	98.11
	Superf	97.81	97.40	97.1
Swin-GA-RF	Dysker	98.14	96.93	97.53
	Koilo	95.57	91.52	93.50
	Metapl	94.58	98.74	96.62
	Parab	100	100	100
	Superf	98.82	100	99.40

**Figure 7:**
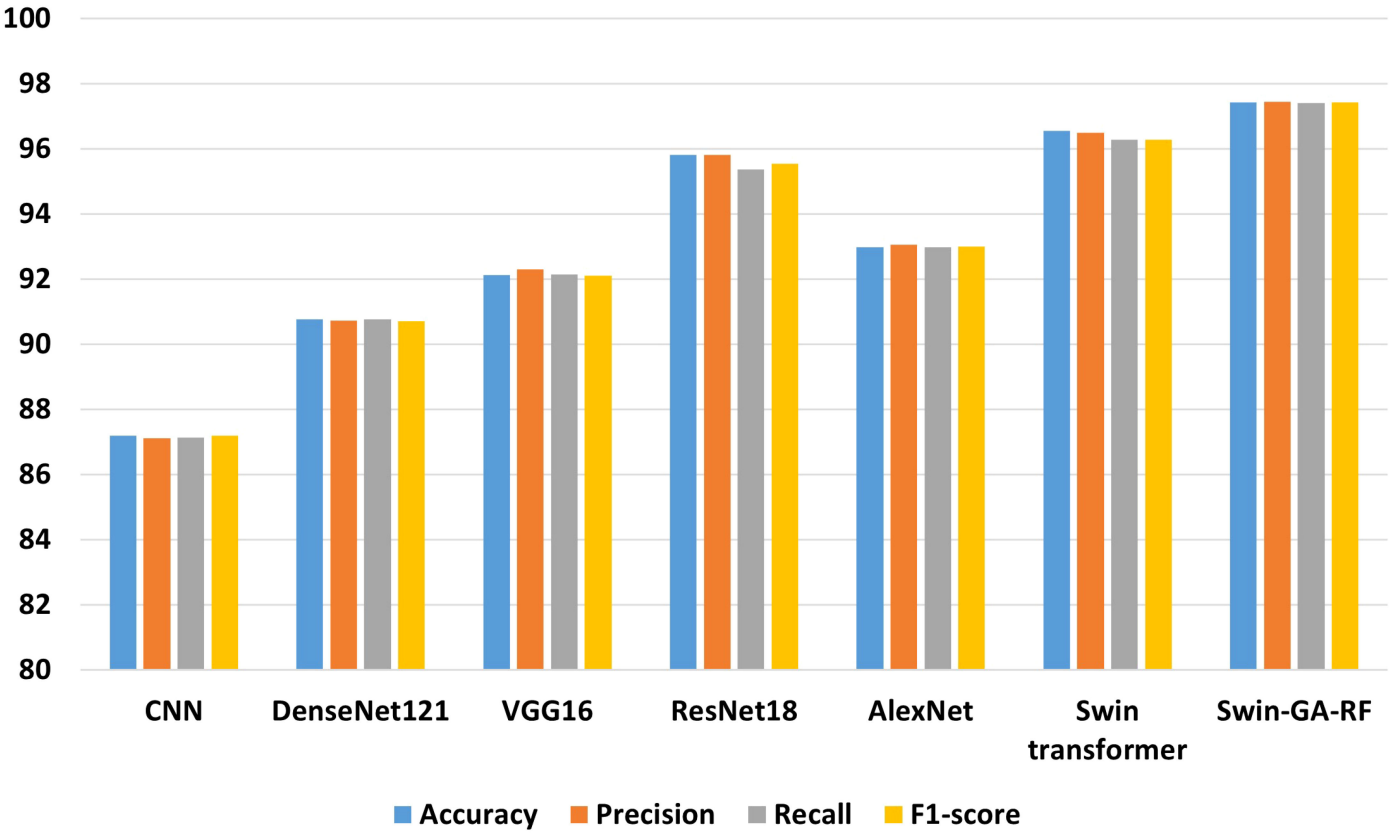
The average of results for models with SDG optimizer for five classes.

**Figure 8:**
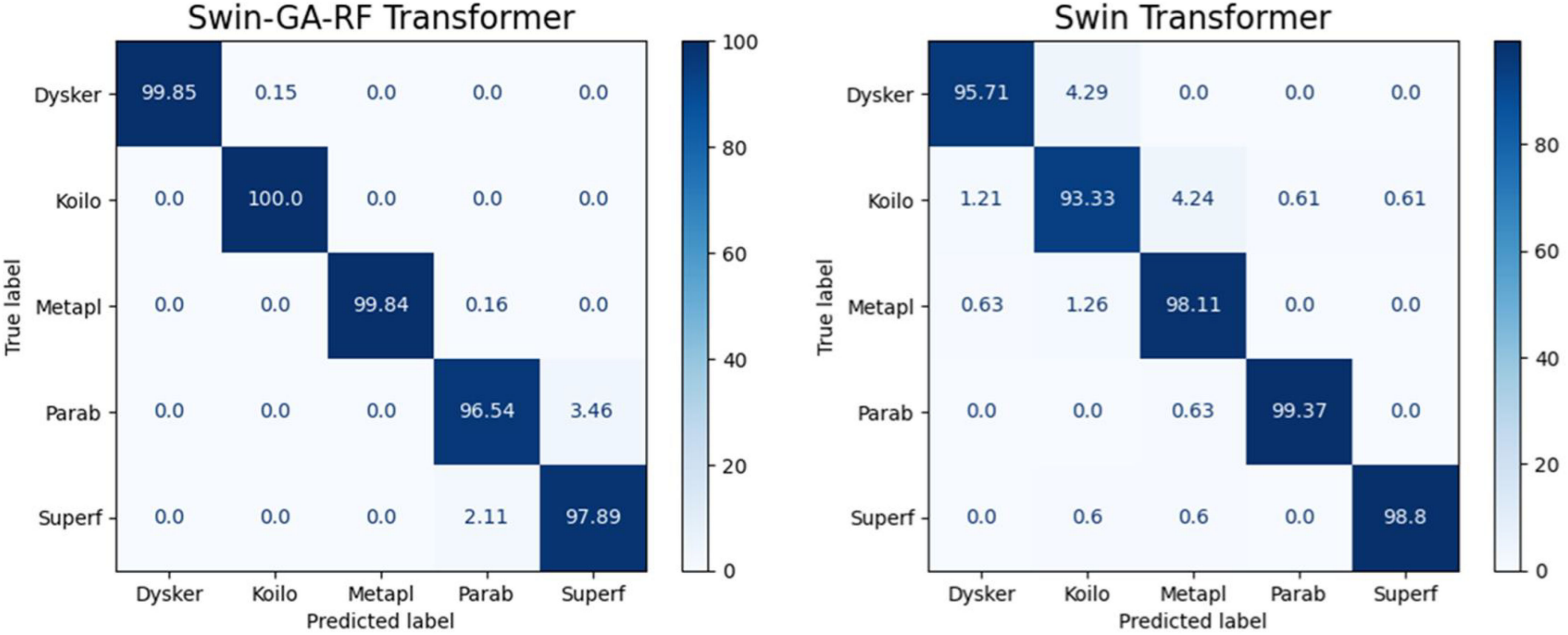
Confusion matrix of Swin and Swin-GA-RF with SDG optimizer for five classes.

**Figure 7** presents the average accuracy, precision, recall, and F1-score of CNN, pre-trained CNN models, Swin Transformer, and Swin-GA-RF models for five classes using Adam optimizer. The Swin Transformer achieves the highest results compared to the pre-trained models. Furthermore, the improved version of the Swin Transformer, referred to as Swin-GA-RF, demonstrates the highest performance with the highest accuracy, F1-score, and improved results at 1.5. The third-highest performance is recorded by ResNet18, with accuracy, precision, recall, and F1-score at 95.813, 95.887, 95.813, and 95.785, respectively. To compare these results, we conducted a detailed comparison between the Swin Transformer and Swin-GARF models in each class, as illustrated in **Figure 8**. Swin-GA-RF improved TP by 3% compared to the Swin Transformer for the Metapl class. Additionally, it records 100 TP for Parab and Superf classes.

### 4.3 The results of applying models with two classes

#### 4.3.1 The results of Adam optimizer

**Table 6** presents the results of each class obtained from different models, including CNN, DenseNet121, VGG16, ResNet18, AlexNet, Swin Transformer, and Swin-GA-RF using Adam to classify two classes of Cervical Cancer with Adam optimizer. We can see that, CNN records the highest recall, and F1-score at 94.44, 96.84, and 95.62, respectively for the normal cells class. CNN records the lowest recall for the abnormal cells class. DenseNet121 records the highest precision, and F1-score at 96.79, 96.84, and 95.17, respectively for normal cells class. VGG16 records the lowest recall at 88.72 for abnormal cells class. ResNet18 records the highest results compared to CNN DenseNet121, VGG16, and AlexNet. The Swin Transformer emerges as a significant improvement over CNN and the pre-trained CNN models, the proposed Swin-GA-RF model outperforms all other models, recording the highest overall performance and exhibits significant enhancements in all classes. It records the highest precision, recall, and F1-score above 99 for each class.

**Table 6:** The results of each class for the models to classify two classes of cervical cancer with Adam optimizer.

Models	Classes	Precision	Recall	F1-score
CNN	abnormal_cells	94.53	89.63	92.02
	normal_cells	93.21	96.49	94.82
DenseNet121	abnormal_cells	90.99	95.43	93.15
	normal_cells	96.79	93.60	95.17
VGG16	abnormal_cells	96.36	88.72	92.38
	normal_cells	92.75	97.73	95.17
ResNet18	abnormal_cells	93.99	95.43	94.70
	normal_cells	96.87	95.87	96.37
AlexNet	abnormal_cells	92.99	93.43	94.70
	normal_cells	93.87	93.87	92.37
Swin transformer	abnormal_cells	98.12	95.73	96.91
	normal_cells	97.15	98.76	97.95
Swin-GA-RF	abnormal_cells	99.32	99.69	99.50
	normal_cells	99.79	99.53	99.66

**Figure 9:**
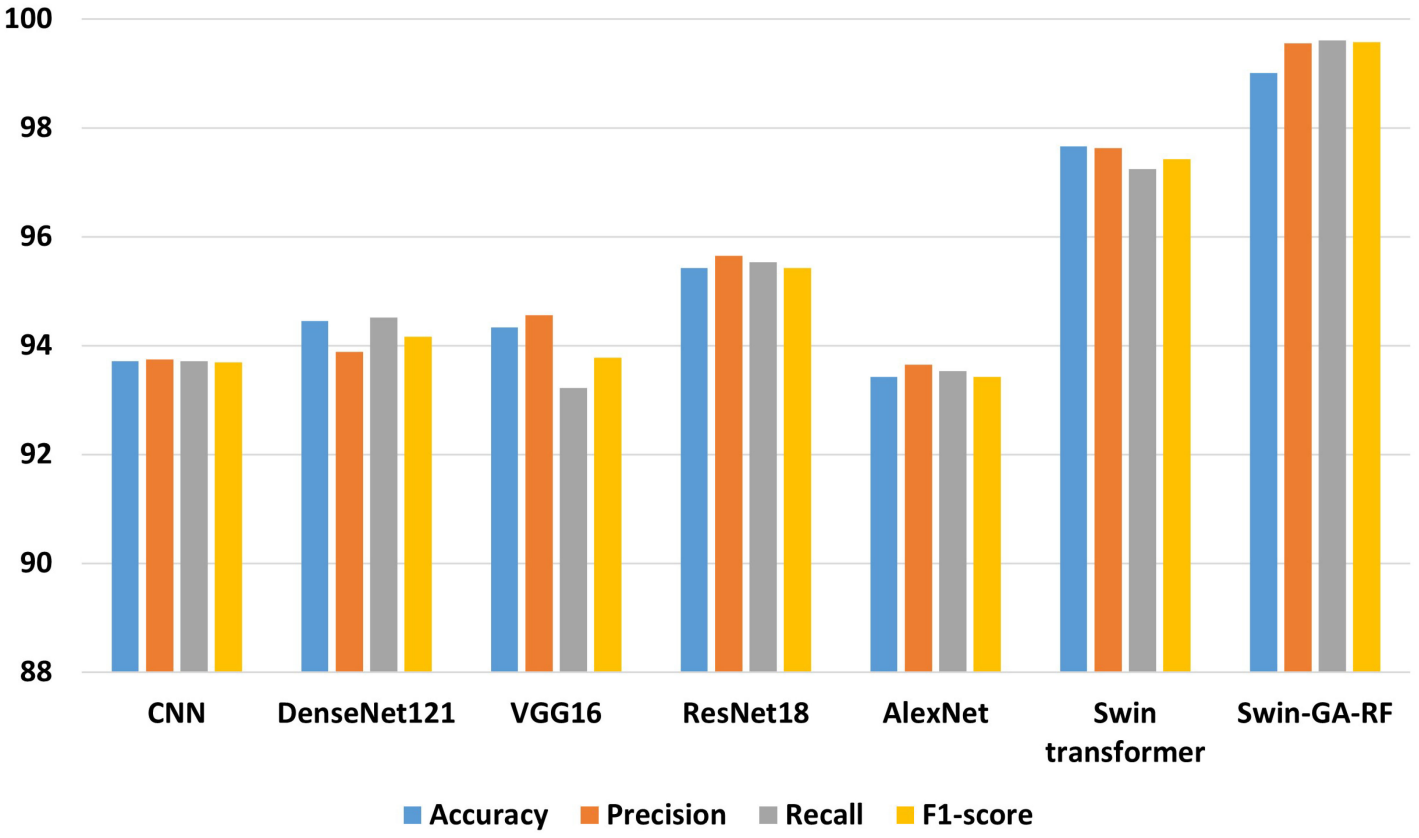
The average of results for models with Adam optimizer for two classes.

**Figure 10:**
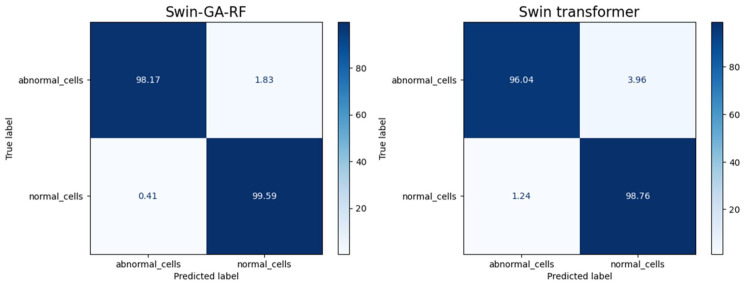
Confusion matrix of Swin and Swin-GA-RF with Adam optimizer for two classes.

**Figure 9** presents the average of accuracy, precision, recall, and F1-score pre-trained CNN models, Swin Transformer, and Swin-GA-RF models using Adam to classify two classes: abnormal cells and normal cells. The Swin Transformer achieves the highest results compared to the pre-trained models. Furthermore, the improved version of the Swin Transformer, referred to as Swin-GA-RF, records the highest performance with the highest accuracy, F1-score, and improved results at 2.5. Specifically, it achieves 99.012 accuracy, 99.555 precision, 99.61 recall, and an F1-score of 99.58. The third-highest performance is recorded by ResNet18. To further compare these results, we conducted a detailed comparison between the Swin Transformer and Swin-GA-RF models in each class, as illustrated in **Figure 10**. Swin-GA-RF improved TP by 4% compared to the Swin Transformer for abnormal cells and normal cells.

#### 4.3.2 The results of SDG optimizer

**Table 7** presents the results of each class obtained from different models, including CNN, DenseNet121, VGG16, ResNet18, AlexNet, Swin Transformer, and Swin-GA-RF using SDG to classify two classes of Cervical Cancer with Adam optimizer. We can see that, CNN records the highest recall, and F1-score at 92.08, 92.87, and 92.47, respectively for the normal cells class. CNN records the lowest recall for abnormal cells class. DenseNet121 records the highest precision, and F1-score at 93.88, 94.66, and 94.96, respectively for normal cells class. VGG16 records the lowest recall at 90.86 for the abnormal cells class. ResNet18 records the highest results compared to CNN DenseNet121, VGG16, and AlexNet. The Swin Transformer emerges as a significant improvement over CNN and the pre-trained CNN models. It exhibits significant enhancements in all classes, the proposed Swin-GA-RF model outperforms all other models, recording the highest overall performance. The Swin-GA-RF model showcases perfect precision, recall, and F1-score, highlighting its ability to accurately classify this type of cervical cancer.

**Table 7:** The results of each class for the models to classify two classes of cervical cancer with SDG optimizer.

Models	Classes	Precision	Recall	F1-score
CNN	abnormal_cells	90.83	90.68	90.25
	normal_cells	92.08	92.87	92.47
DenseNet121	abnormal_cells	93.52	92.38	92.94
	normal_cells	93.88	94.66	94.27
VGG16	abnormal_cells	90.70	92.12	90.86
	normal_cells	94.58	93.39	94.96
ResNet18	abnormal_cells	95.58	94.82	95.69
	normal_cells	95.53	95.73	95.13
AlexNet	abnormal_cells	93.81	92.33	94.60
	normal_cells	93.95	95.25	92.75
Swin transformer	abnormal_cells	96.91	97.65	97.77
	normal_cells	97.70	96.49	97.09
Swin-GA-RF	abnormal_cells	98.13	98.04	98.07
	normal_cells	98.35	98.76	98.05

**Figure 11:**
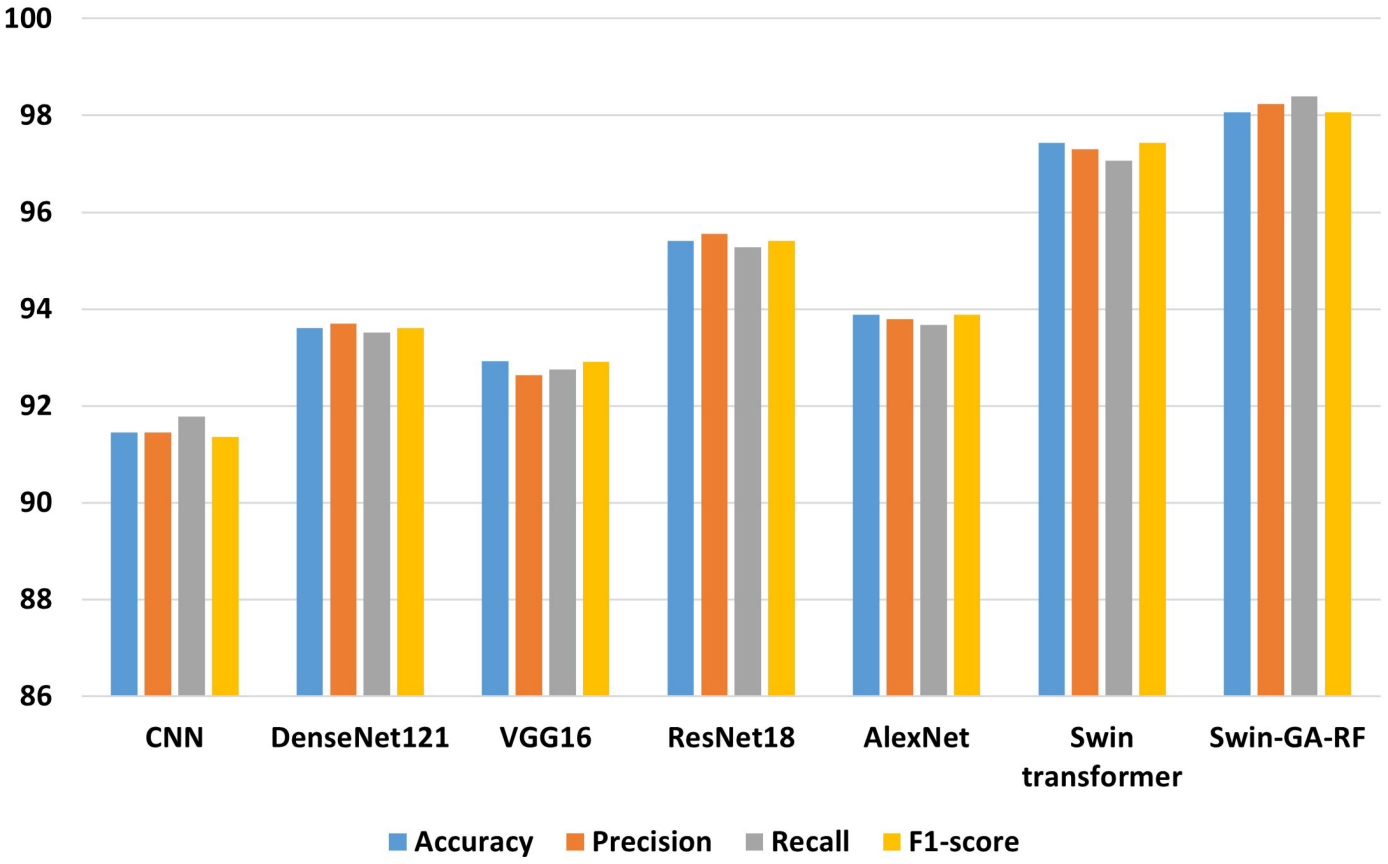
The average of results for models with SDG optimizer for two classes.

**Figure 12:**
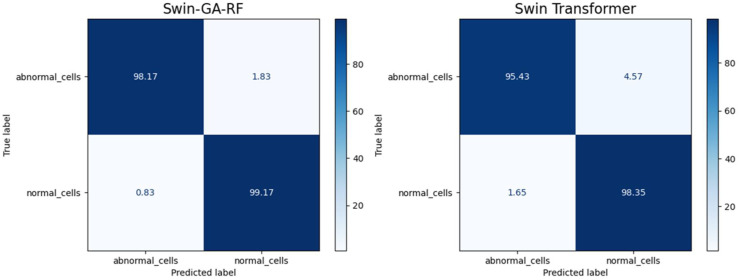
Confusion matrix of Swin and Swin-GA-RF with SDG optimizer for two classes.

**Figure 11** presents the average of accuracy, precision, recall, and F1-score pre-trained CNN models, Swin Transformer, and Swin-GA-RF models using SDG to classify two classes: abnormal cells and normal cells. We can observe that the Swin Transformer achieves the highest results compared to the pre-trained models. Furthermore, the improved version of the Swin Transformer, referred to as Swin-GA-RF, achieves the highest performance with the highest accuracy, F1-score, and improved results at 1.5. It records an accuracy of 98.06, a precision of 98.240, a recall of 98.400, and an F1-score of 98.06. Both DenseNet121 and AlexNet exhibit identical results, with accuracy at 93.605, and 93.88, respectively. The third-highest performance is recorded by ResNet18. To compare these results, we conducted a detailed comparison between the Swin Transformer and Swin-GA-RF models in each class, as illustrated in **Figure 12**. Swin-GA-RF improved TP by 3% compared to the Swin Transformer for abnormal cells and normal cells.

### 4.4 Discussion

#### 4.4.1 The best models

**Figure 13:**
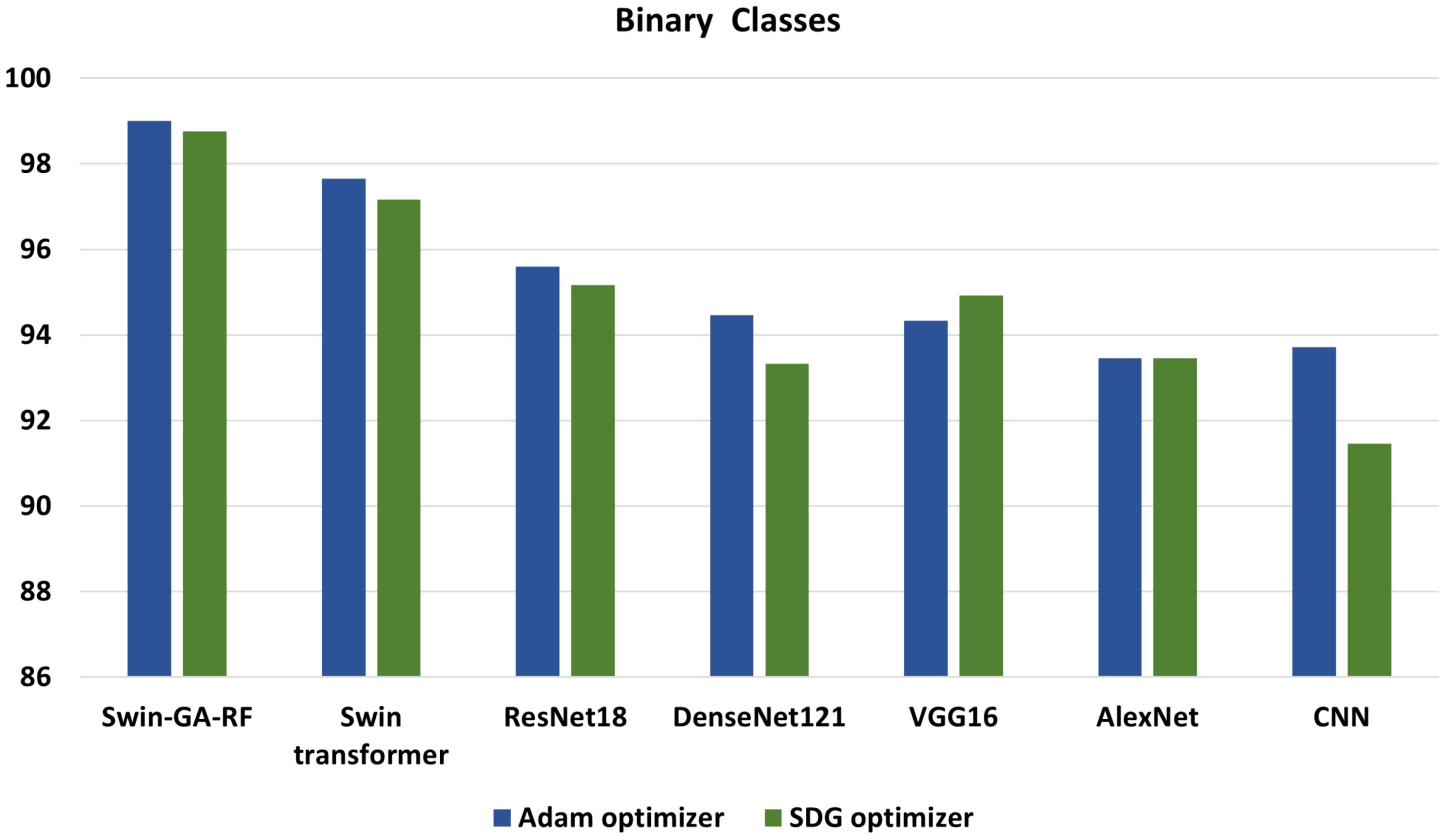
Best models for binary classes.

**Figure 14:**
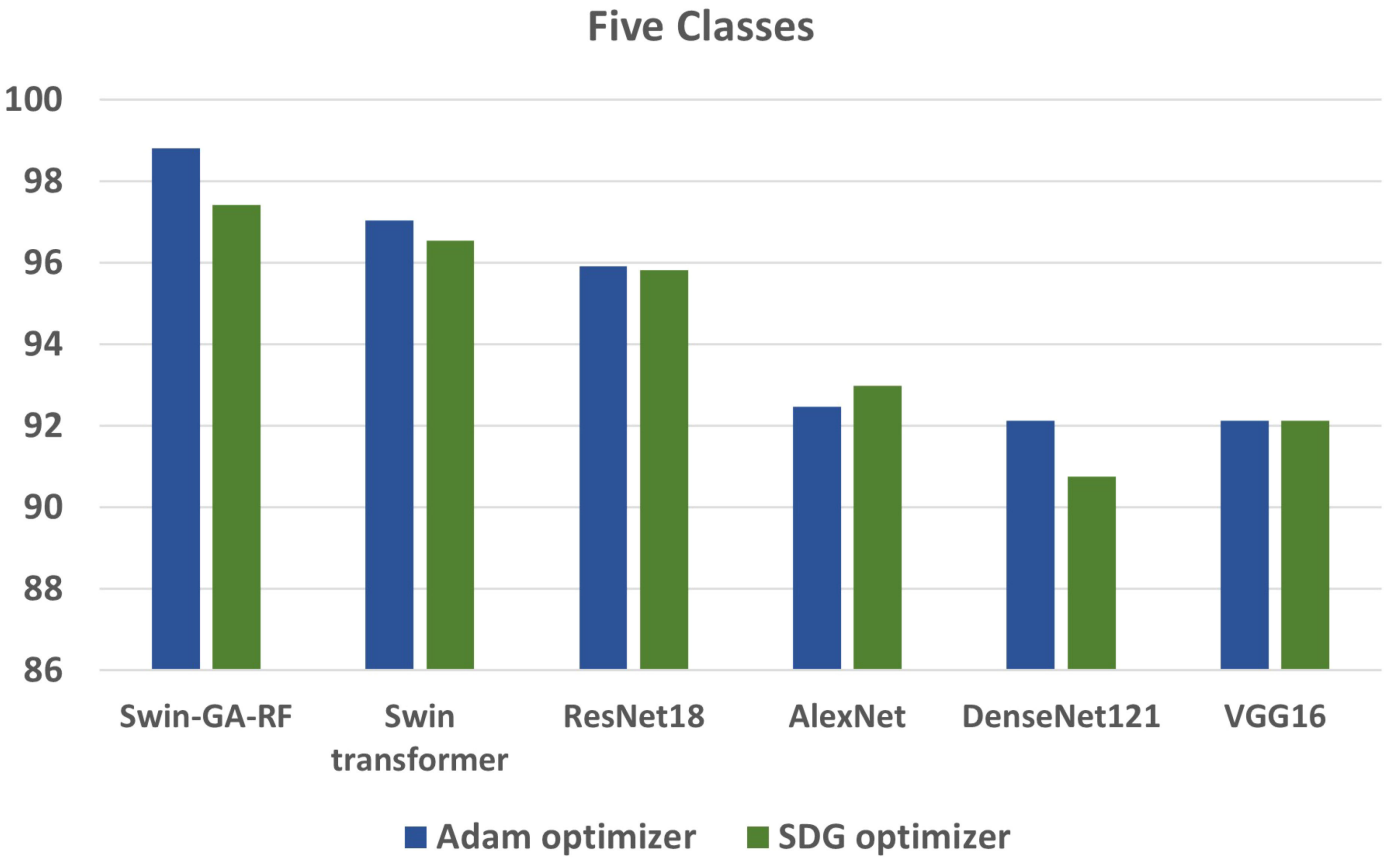
Best models for five classes.

We conducted various experiments to compare the proposed model with different models, including Swin Transformer and pre-trained CNN models, using two optimization methods: SGD and Adam. Additionally, we classified cervical cells in Pap smear images into two classes (abnormal cells and normal cells) and five classes (Dysker, Koilo, Metapl, Parab, and Superf). From **Figure 13** and **Figure 14**, we can observe that the proposed model (Swin-GA-RF) records the highest performance compared to other models. Also, the results using Adam Optimizer have better performance compared to SDG optimizer. **Figure 14** shows a comparison of the best models between two optimizers for five classes. We can see that Swin-GA-RF achieves the highest accuracy at 98.808 with the Adam optimizer, while DenseNet121 has the lowest accuracy at 90.764 with the SGD optimizer. The Swin transformer records the second-highest accuracy at 97.044 for the Adam optimizer.

#### 4.4.2 Statistical analysis

To ensure a rigorous and comprehensive statistical analysis, we employed well-established methodologies in our study. We utilized The Friedman test, Derrac et al. (46), which was chosen due to its suitability for comparing multiple related samples. This non-parametric test allowed us to thoroughly evaluate the presence of significant variations among the models, enabling us to determine whether there were noteworthy differences based on the observed data. The test shows if there is a significant difference between the observed data. Following the Friedman test, we conducted the Nemenyi test as a *post-hoc* analysis to extract the ranks of each model. This additional step provided a deeper understanding of the differences between the models by facilitating multiple comparisons Brown and Mues (47).

**Figure 15:**
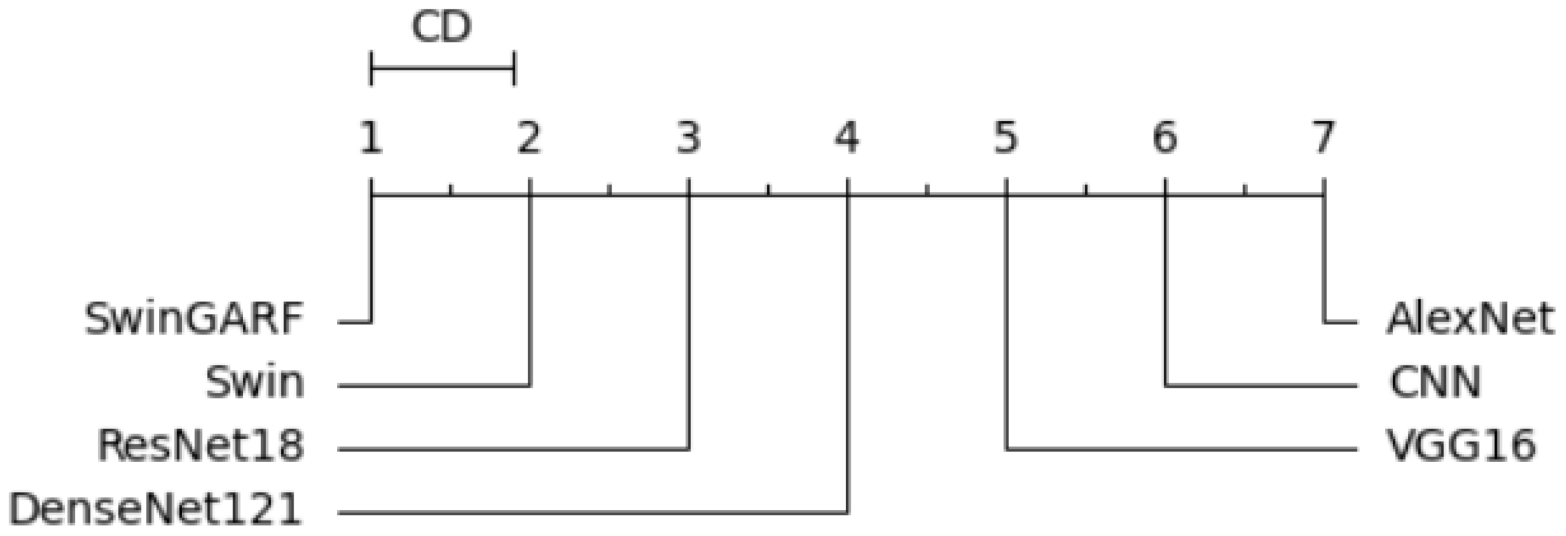
Critical difference diagram.

Specifically, the Nemenyi test allowed us to identify specific pairs of models that exhibited statistically significant differences in their performance, providing valuable insights into their relative strengths and weaknesses. The Nemenyi test represents the difference in terms of critical difference. To enhance the interpretability of the results, we created a critical difference diagram (CD diagram) as depicted in **Figure 15**. The CD diagram, a widely utilized visualization tool in multiple comparison analyses, played a crucial role in highlighting the significant differences among the models. By displaying the average ranks of the models, it provided a clear and concise representation of their performance disparities. Notably, the CD diagram helped identify which pairs of models showed statistically significant differences, further aiding in the comprehensive understanding of their relative performances. The CD diagram offers a concise way to interpret the results of multiple comparison analyses.

#### 4.4.3 Comparison with existing state-of-the-art methods

In this study, we proposed Swin-GA-RF to classify cervical cancer. **Table 8** provided a comparison to the existing studies and the proposed model for classifying cervical cancer. As can be seen, studies are based on different CNN architectures, ViT transformers and do not use Swin transformer and optimization feature extraction methods. We can see that the Swin-GA-RF achieved the highest accuracy in each class case. The authors in AlMubarak et al. (9) Plissiti et al. (10) Alsubai et al. (11) Manna et al. (16) Pramanik et al. (17) Chen et al. (19) to classify five classes of cervical cancer. In AlMubarak et al. (9), the authors applied a hybrid method and recorded an accuracy of 80.72. In Plissiti et al. (10), SVM was utilized, resulting in an accuracy of 94.44. In Alsubai et al. (11), CNN achieved an accuracy of 91.13.

**Table 8:** Comparison with literature studies.

Papers	Models	classes	Swin Transformer	Optimized Feature extraction	Performance
AlMubarak et al. (9)	hybrid method	Five	NO	NO	80.72
Plissiti et al. (10)	SVM	Five	NO	NO	94.44
Alsubai et al. (11)	CNN	Five	NO	NO	91.13
Win et al. (48)	bagging ensemble	Five	NO	NO	94.09
Manna et al. (16)	ensemble-based model	Five	NO	NO	97.55
Pramanik et al. (17)	ensemble-based model	Five	NO	NO	96.96
Deo et al. (49)	CerviFormer	Five	NO	NO	93.70
Chen et al. (19)	CNN with loss	five	NO	NO	96.18
Ghoneim et al. (18)	CNN-ELM	Two	NO	NO	91.20
Manna et al. (16)	ensemble-based model	Two	NO	NO	98.55
Win et al. (48)	bagging ensemble	two	NO	NO	98.27
Pacal and Kılıcarslan (50)	ViT transformer	Five	No	No	91.93
Yaman and Tuncer (20)	ResNet-152	Five	No	No	94.89
Ravindran et al. (51)	SE-ResNet152	–	No	Yes	97.68
Win et al. (48)	RF	Five	NO	NO	95
Win et al. (48)	RF	two	NO	NO	97
Maurya et al. (52)	ViT-CNN	Five	NO	NO	97.65
Li et al. (53)	VTCNet	Five	NO	NO	98.02
Our work	Swin-GA-RF	Five	NO	NO	98.808
Our work	Swin-GA-RF	Two	NO	NO	99.012

Some authors used advanced methods of DL and ensemble learning. For example, ensemble learning models applied in Manna et al. (16) Pramanik et al. (17) Win et al. (48). In Manna et al. (16), an ensemble-based model achieved an accuracy of 98.55. In Win et al. (48), a bagging ensemble method achieved an accuracy of 98.27. In Win et al. (48), a bagging ensemble method attained an accuracy of 94.09. In Manna et al. (16) Pramanik et al. (17), ensemble-based models recorded accuracies of 97.55 and 96.96, respectively. Other applied ViT transformer Pacal and Kılıcarslan (50) recorded 91.93 accuracy. Hybrid models such as ViT-CNN ensemble based on VIT and CNN recorded 97.65 accuracy Maurya et al. (52). SE-ResNet152 is transformer learning that was optimized by DHO and recorded 97.68 accuracy Ravindran et al. (51). In Li et al. (53), VTCNet mobed that combined CNN-SPPF and ViT and recorded 98.02 accuracy. In Deo et al. (49), CerviFormer achieved an accuracy of 93.70. In Chen et al. (19), CNN with loss recorded an accuracy of 96.18. In Ghoneim et al. (18), CNN-ELM achieved an accuracy of 91.20. We can see that the Swin-GA-RF achieved the highest accuracy in each class case.

## 5 The practical implications and potential applications of the proposed methodology

The proposed methodology in our research study has several practical implications and potential applications in the field of cervical cancer diagnosis and beyond. Some of these implications and applications include:

Improved Cervical Cancer Diagnosis: The primary practical implication of our methodology is the potential for significantly improving the accuracy of cervical cancer diagnosis. By leveraging the advanced features of the Swin Transformer, genetic algorithm-based feature selection, and a random forest classifier, our model can effectively classify cervical cells in Pap smear images.
Enhanced Screening Efficiency: Our methodology has the potential to enhance the efficiency of cervical cancer screening programs. This targeted approach can optimize the utilization of healthcare resources and reduce the burden on healthcare systems.
Support for Healthcare Professionals: The proposed methodology can serve as a valuable tool for healthcare professionals involved in cervical cancer diagnosis. By providing a reliable and automated classification system. This support can lead to improved workflow management and better allocation of resources.
Generalizability to Other Medical Imaging Tasks: The combination of the Swin Transformer, genetic algorithm-based feature selection, and a random forest classifier has the potential to be extended to other medical imaging domains. The methodology can be applied to various image-based diagnostic tasks, such as the classification of different types of cancer cells or the detection of other diseases. thereby benefiting a wider range of diagnostic processes.

Finally, it is important to note that while our study focuses on cervical cancer diagnosis, the practical implications and potential applications of our methodology extend beyond this specific domain. The combination of advanced deep learning techniques, feature selection algorithms, and classification models can be adapted and applied to other medical imaging tasks.

## 6 Conclusion

Women’s cervical cancer is frequently fatal, so early detection is crucial to reducing the number of cases. This paper proposes a novel approach to enhancing classification performance by leveraging a combination of techniques, including Swin Transformer, GA feature selection, and replacing the softmax layer with Random Forest. To propose Swin-GA-RF, the Swin Transformer is employed to capture both local and global contextual information from images. Then, we extracted feature representation from the Swin transformer, then A genetic algorithm-based feature selection was used to determine the best feature set from the extracted features, then the SoftMax classifier was replaced by random forest to enhance accuracy. we applied different image augmentation including flipping, cropping, and resizing to enhance the quality of the images. Through extensive experiments, we compared the performance of the proposed Swin-GA-RF model with other models, including the Swin Transformer, CNN, and pre-trained CNN models, using two optimizer methods, SGD and Adam. The results demonstrate that Swin-GA-RF, particularly when utilizing the Adam optimizer, achieved the highest performance in both binary and five-class classification tasks. For binary classification, it achieved an accuracy, precision, recall, and F1-score of 99.012, 99.015, 99.012, and 99.011, respectively. In the five-class classification, it achieved an accuracy, precision, recall, and F1-score of 98.808, 98.812, 98.808, and 98.808, respectively.

While the proposed Swin-GA-RF approach shows promising results, it is essential to acknowledge its limitations. Firstly, The detection and classification were performed at the cell level, which may not fully represent the complexity of cancerous lesions at the tissue or organ level. Additionally, the dataset used in the study is large and contains various classes that are closely related, posing challenges in accurately distinguishing between them. The overlapping features among classes can lead to misclassifications or lower accuracy rates, particularly in distinguishing closely related types of cervical cancer. Future work in this area could focus on expanding the dataset to include a broader range of cervical cell abnormalities, exploring additional optimization techniques for the Swin-GA-RF model, and conducting further comparative analyses with other state-of-the-art approaches. Additionally, efforts can be made to address the practical limitations by improving dataset collection, and data preprocessing techniques, and optimizing the model for efficient deployment in real-world scenarios.

## Data availability statement

The original contributions presented in the study are included in the article/supplementary material. Further inquiries can be directed to the corresponding author.

## Author contributions

MAA: Writing – original draft, Writing – review & editing, Methodology, Validation. NE-R: Investigation, Methodology, Writing – original draft, Writing – review & editing. SAla: Writing – original draft, Writing – review & editing, Software. HE: Writing – original draft, Writing – review & editing, Investigation. SAlh: Writing – original draft, Writing – review & editing, Methodology. HS: Conceptualization, Data curation, Formal analysis, Funding acquisition, Investigation, Methodology, Project administration, Resources, Software, Supervision, Validation, Visualization, Writing – original draft, Writing – review & editing.
